# Kinetic and hydrogen production analysis in the sequential valorization of a *Populus* clone by cold alkaline extraction and pyrolysis

**DOI:** 10.1038/s41598-024-52052-0

**Published:** 2024-01-17

**Authors:** S. Lozano-Calvo, J. M. Loaiza, J. C. García, R. Tapias, F. López

**Affiliations:** 1 Research Centre for Technology of Products and Chemical Processes (PRO2TECS), Department of Chemical Engineering, University of Huelva, Av. 3 de Marzo S/N, 21071 Huelva, Spain; 2 Department of Forest Engineering, University of Huelva, Huelva, Spain

**Keywords:** Energy science and technology, Engineering

## Abstract

This work employed a two-step biorefining process, consisting of a hemicellulose-rich liquor production through ultrasound-assisted cold alkaline extraction (CAE), followed by thermochemical treatment of the resultant solid phase. The post-CAE solid phase’s pyrolytic potential was assessed by application of thermogravimetric analysis (TGA) and Friedman’s isoconversional method, and also from hydrogen production. The solid phases remaining after the CAE treatment were more reactive than the untreated raw material. Notably, the alkali concentration employed in the first step was the individual variable most pronounced influence on their activation energy (*E*_a_). Thus, at a degree of conversion α = 0.50, *E*_a_ ranged from 109.7 to 254.3 kJ/mol for the solid phases, compared to 177 kJ/mol for the raw material; this value decreased with rising glucan content. At maximal degradation, the post-CAE solid phases produced up to 15.57% v/v more hydrogen than did the untreated raw material.

## Introduction

Economic growth and expansive economies have led to an increasingly poorer environmental quality in many countries, especially as regards ecological systems, biodiversity and human health. Economic development is closely related to energy consumption^1^ and, at present, 81% of all primary energy is obtained from fossil fuels, 5% from nuclear fission and only 14% from renewable sources^2^. As a result, atmospheric CO_2_ levels continue to rise steadily. In fact, they have risen by 42% since 1950 and worsened the global problem posed by climate change^3^. In response to this pressing challenge, international institutions have adopted initiatives such as the Paris Agreement, by which countries such as United Kingdom, Spain, France and New Zealand have committed to be carbon–neutral by 2050, and others such as Sweden and Norway expect to meet this goal by 2045 and 2030, respectively^4^. Because societies can hardly be sustainable unless they use low-carbon fuels^5^, growing concern with dramatic climate events and the exhaustion of fossil fuels has emerged over the last few decades that has boosted a search for renewable sources of energy on a global scale^6^.

Biomass is a clean, sustainable choice for a smooth transition from oil-based to biorenewable energy^7,8^. Lignocellulosic biomass is especially effective as a renewable resource because it is widely available in vast amounts, and also because its chemical components are highly suitable and its production does not compete with that of food^8,9^. As a result, second-generation biofuels are being increasingly obtained from lignocellulosic biomass for the production of renewable energy^10^.

Biorefining lignocellulosic materials allows biomass to be fractioned into its three main precursors (cellulose, hemicellulose and lignin) and the precursors to be processed in order to obtain products with a high added value^11^. One of the treatments used for this purpose is cold alkaline extraction, which is especially advantageous in operational and environmental terms. Also, however, it is subject to one major disadvantage: the optimum operating conditions depend on the particular type of biomass to be treated. Thus, CAE is usually much more efficient with grass than it is with wood^12^ and the latter usually requires additional measures for efficient processing^13^. Also, CAE removes both hemicelluloses and polyphenols^14^. The potential applications of the hemicellulosic franction are extensive and have been explored by numerous researchers. For instance, sugar extracted from hemicellulose can subjected to biological conversion for biofuel production, such as ethanol; utilized in chemical processes for the production of substances like xylitol or lactic acid; or chemical conversion produces furfural, 5-hydroxyfurfural, and levulinic acid, crucial precursors for synthesizing various organic derivatives^15^. In addition, the CAE decreases the lignin content of the material and leading to a reduced activation energy upon thermochemical treatment as a result of the polyphenol fraction consisting largely of benzene rings —which possess increased *E*_a_ values and thermal stability^16^.

Thermochemical treatments of lignocellulosic biomass provide a promising technology for producing fuels with a high added value^17^. Highlighting pyrolysis as the most popular and versatile process^18^ for the efficient production of bio-coal, bio-oil and pyrolytic gas (a mixture of carbon monoxide and dioxide, hydrogen, light hydrocarbons and small amounts of other compounds)^19^. Pyrolysing lignocellulosic biomass affects its three main components (cellulose, hemicellulose and lignin)^20^ and involves a number of chemical reactions the span three steps, namely: (1) evaporation of moisture; (2) primary degradation of biomass (200–400 °C); and (3) side reactions^21^. A number of studies have examined the pyrolysis of various types of lignocellulosic biomass and their individual fractions with a view to better understanding their rate and extent of thermal degradation, the products formed and their proportions^22^.

Pyrolysing biomass is also one way of obtaining hydrogen (H_2_) from renewable resources^23^. Hydrogen is one of the major energy vectors for decarbonization strategies in Europe^24^ and pyrolysis produces not only gaseous hydrogen, but also additional hydrogen formed in water–gas exchange reactions with carbon monoxide^25^. Hydrogen production is influenced by several variable, and it has been observed that as the pyrolysis temperature rises, the output of gaseous products increases while the formation of biochar and bio-oil decreases^26^. Thus, a temperature of 750 °C produces about 50% of hydrogen, whereas one of 500 °C gives only about 35%^25^. This is a result of the gas forming in very small amounts in primary reactions relative to tar cracking, reforming and coal decomposition at increased temperatures^27,28^.

Hydrogen production also increases with increasing proportion of cellulose and hemicelluloses, but not lignin^29,30^. In fact, hydrogen forms largely from the cellulose fraction^31^. Ligneous biomass has been found to provide more hydrogen than agricultural residues^32^. Thus, Loaiza et al.^33^ found extracting hemicelluloses from *Eucalytpus urograndis* by pyrolysis to provide solids whose pyrolysis led to a decreased activation energy and increased hydrogen production (6–8%) relative to the untreated, raw material.

The lignocellulosic biomass used in this work was obtained from clone AF2 of the genus *Populus* (viz., *Populus nigra x Populus deltoides*). *Populus* is a fast-growing plant that affords short, highly productive rotation^34^. Also, it is especially well adapted to Mediterranean areas with cold winters^35^ and adequate soil moisture^36^, so its productivity can be affected by climate change^34^. This has promoted the use of hybrid clones capable of growing under suboptimal conditions^37^. Some studies have shown clone AF2 of *Populus* to outperform others under conditions of limited water availability and long droughts^38^.

The main aim of this work was to optimize exploitation of clone AF2 by using a two-step biorefining scheme involving ultrasound-assisted CAE and the subsequent pyrolysis of the resulting solid phase. The degree of conversion was assessed by thermogravimetric analysis (TGA) and non-isothermal kinetic parameters were determined by using Friedman’s differential isoconversional method. Also, the kinetics of the pyrolytic process was modelled in terms of *E*_a_ in order to maximize hydrogen production, which was quantified as a function of the operating conditions of the ultrasound-assisted CAE treatment.

## Materials and methods

### Characterization of the raw material

The lignocellulosic biomass used was from clone AF2 of *Populus* and collected from plantations on La Rabida Campus (University of Huelva, Spain). The collected material was cold ground to obtain chips approximately 1–3 cm long and 0.5 cm wide.

The raw material was previously characterized by some members of our group in terms of moisture^39^, solubility in 1% soda^40^, solubility in hot water^41^, ethanol–acetone extractives^42^ and ash^43^. In addition, the material was subjected to acid hydrolysis in two steps involving treatment with 72% sulphuric acid at 30 °C for 1 h to cleave polysaccharides into oligomers and 4% sulphuric acid at 121 °C for 1 h to convert the oligomers into monomers. Monomeric sugars and Klason lignin were determined by HPLC according to^44^ and^45^, respectively. The results of the previous determinations are summarized in Table 1.

**Table 1:** Percent composition of the raw material.

1% NaOH extractives	22.3 ± 1.95
Hot water extractives	4.22 ± 0.12
Ethanol–acetone extractives	2.8 ± 0.15
α-Cellulose (glucan)	40.3 ± 0.85
Klason lignin	25.1 ± 0.16
Xylan	19.3 ± 0.09
Arabinan	0
Acetyl groups	0.7 ± 0.02
Ash	1.14 ± 0.04

### Ultrasound-assisted cold alkaline extraction

A CAE treatment was used to maximize extraction of hemicelluloses into the liquor and ensure that they would remain stable for potential subsequent processing. The treatment was conducted in a Power Sonic Series 500 bath, using a water modulus (i.e., a liquid/solid ratio) of 15 kg water/ kg raw material on a dry basis, an ultrasound power of 120 W, an alkali concentration of 80–120 g/L, a temperature of 20–40 °C (temperature was kept constant by cooling the bath) and a treatment time of 30–90 min. The process was followed by filtration to separate the liquor from the remaining solid phase.

The CAE liquor was neutralized with 37% v/v hydrochloric acid, and the hemicellulose fraction precipitated with ethanol. The solid phase was neutralized with acetic acid, rinsed in water, centrifuged and dried at room temperature to determine moisture as described in Section "Characterization of the raw material" and calculate CAE yield by weighing. The solid was additionally hydrolysed in two steps as described in Section "Characterization of the raw material" in order to determine its composition.

The previous conditions were used in combination with a central composite factor design and response surface analysis^46^ to optimize the CAE treatment by examining the influence of the independent variables of the process (viz., alkali concentration, temperature and treatment time) on the composition of the post-CAE solid phase (viz., glucan, xylan and Klason lignin), the activation energy of the solid and its pyrolytic hydrogen production capacity.

The factor design used allowed second-order polynomials to be established whose coefficients were a measure of the statistical significance of the influence of each independent variable on the dependent variables. For easier comparison, the independent variables were normalized by using the following equation:

1 $${{\text{X}}}_{{\text{n}}}=2*\frac{{\text{X}}-\overline{{\text{X}}} }{({{\text{X}}}_{{\text{max}}}-{{\text{X}}}_{{\text{min}}})/2}$$

where *X*, $\overline{{\text{X}} }$, *X*_max_ and *X*_*min*_ are the absolute, mean, maximum and minimum value, respectively, of the variable concerned. The design was applied at three different alkali concentrations (80, 100 and 120 g/L), temperatures (20, 30 and 40 °C) and treatment times (30, 60 and 90 min). It encompassed three groups of experimental points, namely: 2^*n*^ points for the factor design proper, 2*n* axial points and *n*_c_ central points. Therefore, the total number of experiments required, given by 2^*n*^ + 2*n* + *n*_c_, was 16.

Experimental data were modelled by fitting the results for the dependent variables to the following polynomial equation:

2 $${\text{Y}} = {\text{a}}_{0} + \sum\limits_{{{\text{i}} = 1}}^{{\text{n}}} {{\text{b}}_{{\text{i}}} {\text{X}}_{{{\text{ni}}}} + \sum\limits_{{{\text{i}} = 1}}^{{\text{n}}} {{\text{c}}_{{\text{i}}} {\text{X}}_{{{\text{ni}}}}^{2} + \sum\limits_{{{\text{i}} = 1,{\text{j}} = 1}}^{{\text{n}}} {{\text{d}}_{{{\text{ij}}}} {\text{X}}_{{{\text{ni}}}} {\text{X}}_{{{\text{nj}}}} } ({\text{i}} < {\text{j}})} }$$

where *X* denotes independent variables and *Y* dependent variables, and the coefficients a_0_, b_*i*_, c_*i*_ and d_*ij*_ were estimated individually for each independent variable.

The independent terms selected were those having a statistically significant coefficient (*p* < 0.05). The conditions imposed on the overall statistics were *R*^2^ > 0.9 and *F*_Snedecor_ > 5. All statistical processing was done with the software Statistica v. 10.0 from Statsoft, Inc. (Tulsa, OK, USA).

### Thermogravimetric analyses

The raw material and the different post-CAE solid phases were subjected to non-isothermal thermogravimetric analysis (TGA) on a TGA/DSC1 STARe System from Mettler Toledo. In each test, an amount of ca. 13 g of sample was heated from 25 to 800 °C at four different rates (5, 10, 15 and 20 °C/min), using nitrogen at a flow-rate of 10 mL/min as inert gas. The experiments were performed in triplicate recorded the average values.

### Friedman’s analytical method

Friedman’s method was used here to examine the kinetics of the pyrolysis reactions and determine their activation energy. The overall kinetics of the biomass pyrolysis process can be represented as follows:

3 $$\frac{d\alpha }{dt}=k\left(T\right)\cdot f(\alpha )$$

where α is the conversion fraction [(*W*_0_–*W*_*t*_)/(*W*_0_–W_∞_)], *W* denoting the mass of sample, and *W*_0_, *W*_*t*_ and *W*_∞_ its initial value, that at time *t* and that at the end of the process, respectively; *f*(α) the differential conversion function; *T* (K) temperature; and k(*T*) the rate constant in the Arrhenius equation [k(*T*) = *A*·exp(− *E*_a_/R*T*), with *A* the pre-exponential factor, *E*_a_ the apparent activation energy and R the universal gas constant (8.314 J/mol·K)]. The reaction rate can be expressed as follows:

4 $$\frac{d\alpha }{dT}=\left(\frac{A}{\delta }\right)\cdot {\text{exp}}\left(-\frac{Ea}{R\cdot T}\right)\cdot f(\alpha )$$

where δ is the linear heating rate (δ = d*T*/d*t*), which is constant.

For convenience, Eq. (4) can be expressed in integral form:

5 $${\int }_{0}^{\alpha }\frac{d\alpha }{f\left(\alpha \right)}=G\left(\alpha \right)=\left(\frac{A}{\beta }\right)\cdot {\int }_{{T}_{0}}^{T}{\text{exp}}\left(-\frac{{E}_{a}}{R\cdot T}\right)dT=\left(\frac{A}{\beta }\right)\cdot p(x)$$

where *G*(α) and *p*(*x*) are the integrals of the conversion function and temperature, respectively. The latter function cannot be solved exactly, so it has to be estimated or integrated numerically^47^.

Some empirical methods allow the kinetic parameters to be calculated from the data for the thermal analysis. Such methods are of either the isoconversional or the fitting type. In this work, we used an isoconversional method to calculate the kinetic parameters for no specific form of the reaction model to enable the accurate determination of the activation energy^48,49^. The most widely used isoconversional method is Friedman’s differential method^50^. In natural logarithmic form, Eq. (4) becomes:

6 $${\text{ln}}\left(\beta \cdot \frac{d\alpha }{dT}\right)={\text{ln}}\left[A\cdot f\left(\alpha \right)\right]-\frac{{E}_{a}}{R\cdot T}$$

Friedman’s method assumes the extent of biomass decomposition to depend solely on the mass loss rate. Therefore, a plot of ln (dα/d*t*) against 1/*T* allows he activation energy to be calculated from the slope (− *E*_a_/R) and conversion from the intercept [ln [A·*f*(α)].

### Modeling and optimization of activation energy

The modeling and optimization of the E_a_ results, as a function of the independent operating variables of the CAE process with ultrasounds, has been conducted following the methodology outlined in section "Thermogravimetric analysis (TGA) of the pyrolytic process".

The experimental design points for the central composite design are consistent with those utilized for modeling and optimizing the composition of the solid phase.

The same procedures, including response surface analysis, normalization of independent variables, analysis of coefficients, statistical assessment of model validity and/or uncertainty, and the selection of statistically significant coefficients, have been applied. It is noteworthy that the results of the dependent variable (E_a_) were obtained in accordance with the description provided in sections "Thermogravimetric analyses" and "Friedman’s analytical method".

### Quantifying hydrogen production in a pyrolytic reactor

The amount of hydrogen resulting from pyrolysis of the lignocellulosic biomass and the CAE solid phases was evaluated using a laboratory-scale reactor. The reactor consisted of a 140 cm length and 10 mm wide quartz tube, within which approximately 2 g of the sample was uniformly introduced and placed in a furnace at a constant temperature for 4 min, using nitrogen at a flow-rate of 150 mL/min as carrier gas. The operating variables of this laboratory-scale reactor are the furnace temperature, the residence time of the sample in the furnace, and the flow-rate of carrier gas. While it is at a laboratory scale, we believe the results would be scalable. The reactor design is an original design from the research group, and efforts have been made to operate it in a manner resembling that of a semi-pilot reactor.

Hydrogen production was measured by collecting samples in Tedlar bags at the reactor outlet. Samples were collected at the highest degradation temperature in each test for 1 min. Four different samples were taken in each experiment to determine the maximum H_2_, which was produced between minutes 2 and 3. These samples were analyzed using a precalibrated MultiRAE IR PGM-54 gas analyser from RAE Systems (San José, CA, USA). All measurements were made in triplicate and averaged to calculate the volume of pyrolytic gas of the analyzed sample and disregarding the carrier gas.

### Ethics approval and consent to participate

The authors declare that this is their original work, and the corresponding manuscript has not been published before and is only submitted to the Journal of Scientific Reports.

## Results

### Ultrasound-assisted cold alkaline extraction of hemicellulose

Previous studies by our group^51^ revealed that using ultrasound in the CAE of clone AF2 of *Populus* resulted in increased extraction of all lignocellulosic fractions but especially those of hemicelluloses and polyphenols. Specifically, extraction of hemicelluloses, measured as xylan, from the raw material into the CAE liquor ranged from 39.8 to 56.7% in the absence of ultrasound and from 44.3 to 60.8% in its presence. Also, the hemicellulose content of the post-CAE solid phase decreased from 60.2–43.3% to 55.7–39.2% of the content in the starting material. On the other hand, the lignin content of the solid increased from 51.5 to 77.7% with sonication to 71.6–98.9% without it. Finally, cellulose was scarcely extracted into the CAE liquor and remained in the solid phase by 80.4–96.2% with ultrasound and by 85.1–98.7% without it.

These results allowed polynomial models relating the dependent variables (glucan, xylan and Klason lignin in the post-CAE solid phase) to the independent variables (alkali concentration, temperature and treatment time) to be developed. Table 2 shows the equations for the models, which were used to predict the effect of each independent variable of the ultrasound-assisted CAE process on the cellulose, hemicellulose and polyphenol contents of the resulting solid phase. Such contents (specifically, those of glucan, xylan and Klason lignin) are referred to their initial levels in the starting, raw material.

**Table 2:** Equations of the polynomial models for the dependent variables in the ultrasound-assisted CAE treatment.

Equation	Adjusted *R*^2^	Adjusted Snedecor’s *F*
$$\begin{aligned} {\text{GL}} = & {87}.0 - {2}.{5}0X_{{\text{A}}} - {1}.{87}X_{T} + {5}.{98}X_{{\text{A}}}^{{2}} \\ - & {4}.0{3}X_{T}^{{2}} - {4}.{39}X_{{\text{A}}} X_{t} - {1}.{6}0X_{{\text{A}}} X_{T} + {1}.{19}X_{t} X_{T} \\ \end{aligned}$$ (8)	0.98	59
$$\begin{aligned} X = & {4}0.{2} - {2}.{28}X_{{\text{A}}} - {2}.{24}X_{t} - {1}.{12}X_{T} + {7}.{23}X_{{\text{A}}}^{{2}} \\ + & {1}.{62}X_{T}^{{2}} - 0.{56}X_{{\text{A}}} X_{t} + {1}.{37}X_{{\text{A}}} X_{T} + 0.{44}X_{t} X_{T} \\ \end{aligned}$$ (9)	0.89	138
$$\begin{aligned} {\text{KL}} = & {66}.{6} - {5}.{96}X_{t} - {2}.{57}X_{T} - {5}.{33}X_{{\text{A}}}^{{2}} + {4}.{58}X_{T}^{{2}} \\ + & {1}.0{4}X_{{\text{A}}} X_{t} + {3}.{28}X_{{\text{A}}} X_{T} - {2}.{34}X_{t} X_{T} \\ \end{aligned}$$ (10)	0.98	114

Dependent variables: GL glucan (%), X xylan (%), KL Klason lignin (%).

Independent variables: *X*_A_ alkali concentration, *X*_*T*_ temperature, *X*_*t*_ treatment time.

The differences between the experimental values and those estimated with the equations never exceeded 10% of the former.

As can be seen from Eq. (8) in Table 2, the alkali concentration was the most influential variable on the glucan content of the post-CAE solid phase. Cellulose extraction into the liquor was maximal at the greatest alkali concentration, temperature and time values studied. As noted earlier, however, glucan was scarcely extracted into the liquor (20.22% at most).

Based on Eq. (9) in Table 2, medium alkali concentrations and temperatures in combination with long treatment times should result in maximal hemicellulose extraction into the CAE liquor. Finally, Eq. (10) suggests that long CAE treatment times should result in increased delignification (i.e., in increased proportions of lignin in the CAE liquor). This prediction is consistent with the results of Methrath Liyakathali et al.^52^, who found the treatment time and sonication to influence the cleavage of lignin–cellulose bonds to an extent increasing with the former variable. Accordingly, lignin extraction into the CAE liquor was especially high in the upper range of treatment times (90 min).

The post-CAE solid phase was rich in cellulose, but poor in lignin by effect of the polyphenol fraction being largely extracted into the liquor. In theory, this must decrease the pyrolysis activation energy since the polyphenol fraction is that with the highest *E*_a_ value^16^. Also, it should result in increased hydrogen production relative to the starting material^53^. These two expectations warranted the present work, where we examined the pyrolytic process as described below.

### Thermogravimetric analysis (TGA) of the pyrolytic process

The CAE treatment provided a highly valorizable liquor by effect of the extraction process being especially selective for hemicelluloses. Also, the treatment provided a solid phase enriched with a cellulose fraction potentially useful for purposes such as producing energy, hydrogen, biofuels, pulp, paper or polyphenol derivatives. In this work, we examined the potential of the process for obtaining energy and a high added value product: hydrogen. Removing most of the hemicellulose fraction and part of the polyphenol fraction decreased the activation energy of the pyrolysis process^16,33^, thereby presumably facilitating it. It should be noted that, as shown for treatments other than CAE, the operating conditions —or rather, their combination—influence the activation energy and kinetics of the thermal treatment^54^.

Because using all 16 points of the experimental design would have been impractical, we selected those corresponding to the lowest and highest extraction of hemicelluloses (viz., 80 g/L, 90 min, 20 °C and 100 g/L, 60 min, 30 °C, respectively) and the lowest and highest extraction of Klason lignin (viz., 80 g/L, 30 min, 20 °C and 80 g/L, 90 min, 40 °C, respectively) in addition to the raw material.

**Figure 1:**
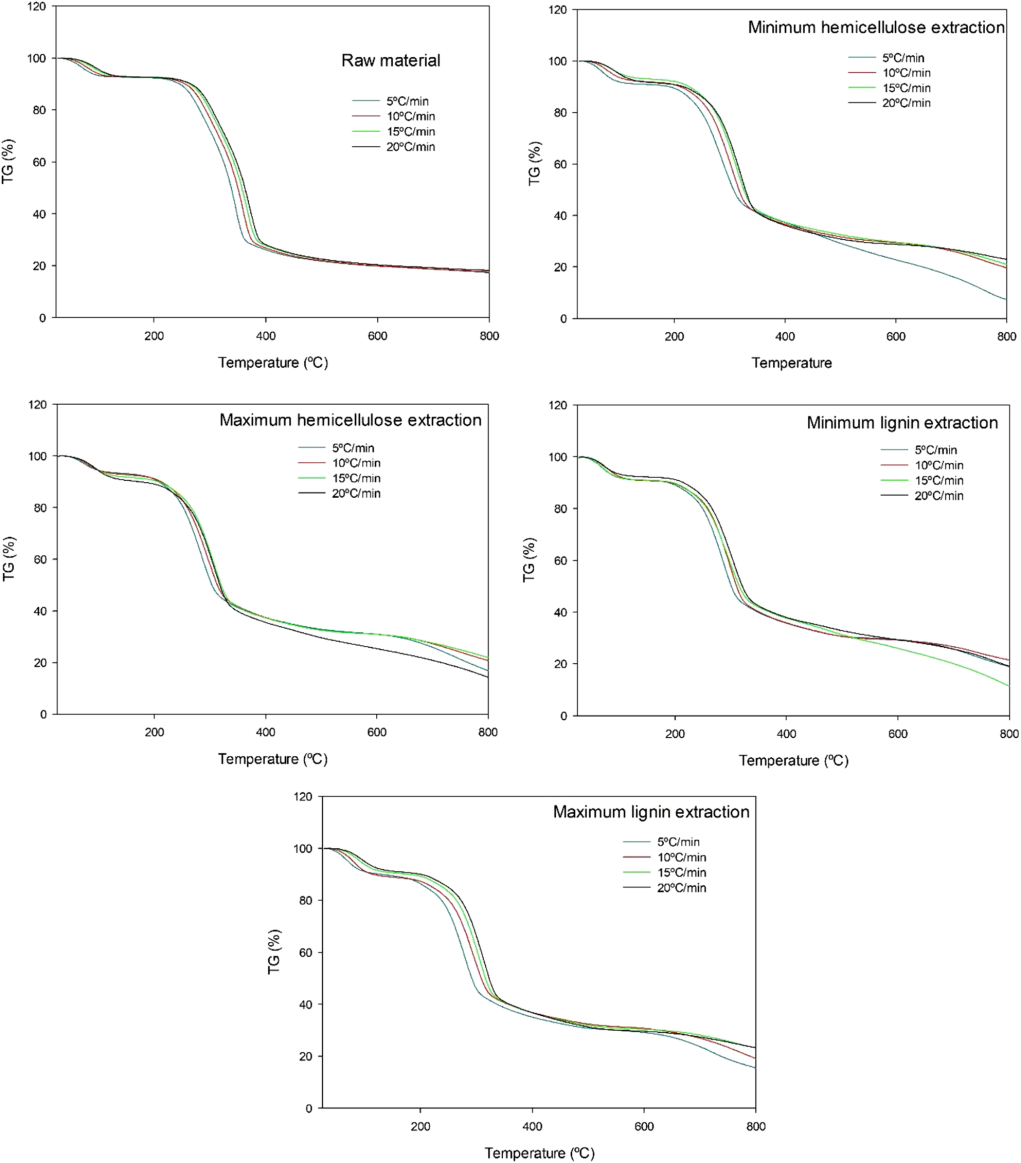
TGA at different heating rates of the raw material and the four post-CAE solid phases.

Figure 1 is a weight loss versus temperature plot. As can be seen, the initial temperature of the second step of process, known as “active pyrolysis”, was higher for the raw material (224 °C) than it was with the lowest and highest extraction of hemicelluloses (172 and 180 °C, respectively) and lignin (177 and 172 °C, respectively). The decreased temperatures for the post-CAE solid phases relative to the raw material can be ascribed to selective extraction of hemicelluloses and polyphenols into the CAE liquor. Also, residual lignin in the resulting solid contained fewer bonds and was thus less thermally stable. The affinity of alkaline treatments for soluble lignin and the cleavage of C–C bonds in polyphenols remaining in the solid fraction was previously observed by other authors^54^. The cleavage of bonds in the lignin fraction decreases its strength and degree of polymerization, thus facilitating hydrolysis of polysaccharides^55^.

Heating the post-CAE solid phase with the highest content in hemicelluloses at 5 °C/min led to an extended loss of fixed carbon that was not considered in calculating the activation energy. Nor was the result for the residue heated at 15 °C/min leading to the lowest extraction of lignin.

### Derivative thermogravimetry (DTG)

**Figure 2:**
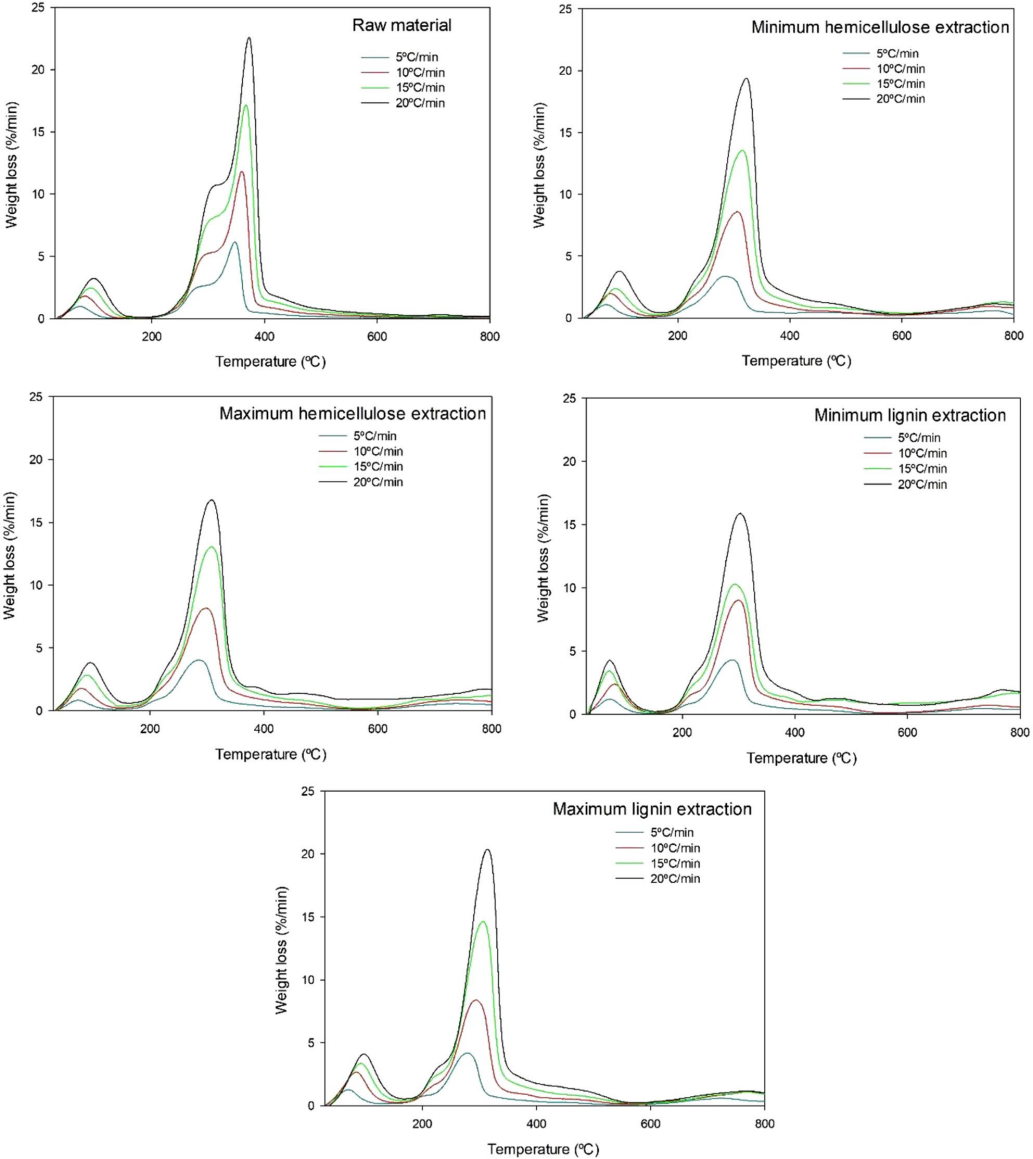
DTG of the raw material and the four post-CAE solid phases.

Figure 2 shows the rate of weight loss (%/min) as a function of temperature (i.e., the DTG curves) for the raw material and the four post-CAE solid phases examined in the previous section. As can be seen, there were three distinct regions typical of biomass pyrolysis corresponding to water evaporation, active pyrolysis ad side reactions^21^. The main pyrolysis step spanned the range 200–400 °C and two sub-steps involving degradation of the cellulose and hemicellulose fractions. Whereas the raw material exhibited two well-defined peaks, the post-CAE solid phases showed a poorly defined peak for hemicellulose degradation. This was a result of structural and compositional differences between the samples directly influencing hemicellulose and cellulose degradation^56^.

Thermal degradation in the active pyrolysis step started at 230 °C in the raw material and at around 190 °C in the post-CAE solid phases, possibly as a result of increased extraction of hemicelluloses and lignin from the latter. Thus, the CAE treatment caused a portion of the polyphenolic fraction to be extracted into the liquor and the rest, which remained in the solid, to break more easily as a consequence of its having a decreased number of C–C bonds —and being less thermally stable as a result^57^.

As can also be seen from Fig. 2, the maximum loss temperature (*T*_max_) was 372 °C for the raw material and lower (302–320 °C) for the post-CAE solid phases. *T*_max_ is known to be inversely related to reactivity^58^. Again, the post-CAE solid phases were less thermally stable than the raw material. Also, *T*_max_ coincided with the main peak in the DTG curves, which was primarily associated with cellulose degradation^55^.

The maximum reaction rate at a heating rate of 20 °C/min was 22.59%/min for the raw material (40.3 wt% glucan), about 19.50%/min for the post-CAE solid phases resulting from the lowest extraction of hemicelluloses and highest extraction of lignin (38.3 and 39.0 wt% glucan, respectively), and in the region of 16.3%/min for those with the highest extraction of hemicelluloses and lowest extraction of lignin (35.0 and 35.7 wt% glucan, respectively). Therefore, it can be observed that the maximum reaction rate increases alongside the glucan content. It can be asserted that the primary degradation, which occurs in this peak of the DTG curves, largely corresponds to the degradation of the cellulose fraction.

### Kinetic analysis of the pyrolytic process

The pyrolysis kinetics of the raw material and the post-CAE solid phases allowed the influence of the independent variables of the treatment on the thermochemistry of the process to be established. The activation energy *E*_a_ at a given degree of conversion α was calculated by applying Friedman’s isoconversional method to the results obtained at the heating rates 5, 10, 15 and 20 °C/min. Table 3 shows the *E*_a_ values thus obtained as the average of at least three determinations each. The relative standard deviation was less than 5% in all instances.

**Table 3:** Activation energy as calculated with Friedman’s isoconversional method at different degrees of conversion for the post-CAE solid phases.

Operating conditions (*X*_A_, *X*_*t*_, *X*_*T*_)			*E*_a_ (kJ/mol)		
			α = 0.25	α = 0.50	α = 0.75
0	0	0	145.7	179.5	285.9
1	1	1	215.4	227.1	139.1
1	1	− 1	152.9	195.3	135.1
1	− 1	1	127.6	158.8	195.4
1	− 1	− 1	131.9	164.4	265.4
− 1	1	1	177.6	153.6	253.3
− 1	1	− 1	165.8	211.9	262.7
− 1	− 1	1	154.8	190.5	167.3
− 1	− 1	− 1	186.8	262.2	255.4
1	0	0	141.1	170.9	282.9
− 1	0	0	174.5	209.6	312.8
0	1	0	133.8	166.1	198.3
0	− 1	0	108.2	145.1	222.9
0	0	1	156.5	183.2	212.3
0	0	− 1	152.9	185.8	290.3

Independent variables: X_A_ alkali concentration, *X*_*T*_ temperature, *X*_*t*_ treatment time.

*E*_a_ for the raw material was 168, 177 and 179 kJ/mol at a degree of conversion of 0.25, 0.50 and 0.75, respectively. As can be seen, the activation energy changed with α, both in the raw material and in the post-CAE solid phases. This was a result of the pyrolysis of such highly complex materials as lignocellulosic biomass being an intricate process involving a number of steps. Some authors have reported an average *E*_a_ value for the whole conversion range whereas others believe that the effective activation energy of lignocellulosic biomass depends on those of decomposition of each fraction^59–61^.

The data in Table 3 were subjected to multiple regression analysis on *E*_a_ as a function of the three independent variables (alkali concentration, temperature, and treatment time) at the different degrees of conversion considered. The equations for the models thus established are shown in Table 4. The coefficients of variation between the predicted and experimental results were all less than 10% and the quadratic regression coefficients, *R*^2^, greater than 0.95. Student’s-*t* values for the individual variables were all greater than 2, so the results were statistically significant at *p* < 0.05.

**Table 4:** Equations of the polynomial models for the activation energy.

Equation	α
$$\begin{aligned} E_{{\text{a}}} = & {137}.{91} - {9}.{18}X_{{\text{A}}} + {13}.{73}X_{t} + {21}.{81}X_{{\text{A}}}^{{2}} - {14}.{95}X_{t}^{{2}} \\ + & {18}.{74}X_{T}^{{2}} + {13}.{5}0X_{{\text{A}}} X_{t} + {12}.0{4}X_{{\text{A}}} X_{T} + {11}.{57}X_{t} X_{T} \\ \end{aligned}$$ (11)	0.25
$$\begin{aligned} E_{{\text{a}}} = & {173}.{91} - {16}.{18}X_{{\text{A}}} + {8}.{3}X_{t} + {18}.{68}X_{{\text{A}}}^{{2}} - {16}.0{1}X_{t}^{{2}} \\ + & {12}.{92}X_{T}^{{2}} + {29}.{62}X_{{\text{A}}} X_{t} + {25}.{57}X_{{\text{A}}} X_{T} \\ \end{aligned}$$ (12)	0.50
$$\begin{aligned} E_{{\text{a}}} = & {276}.{27} - {18}.{69}X_{{\text{A}}} - {16}.{48}X_{t} - {28}.{84}X_{T} + {23}.{98}X_{{\text{A}}}^{{2}} \\ - & {63}.{23}X_{t}^{{2}} - {22}.{54}X_{T}^{{2}} - {4}0.{84}X_{{\text{A}}} X_{t} + {23}.{22}X_{t} X_{T} \\ \end{aligned}$$ (13)	0.75

Independent variables: X_A_ alkali concentration, *X*_*T*_ temperature, *X*_*t*_ treatment time.

**Figure 3:**
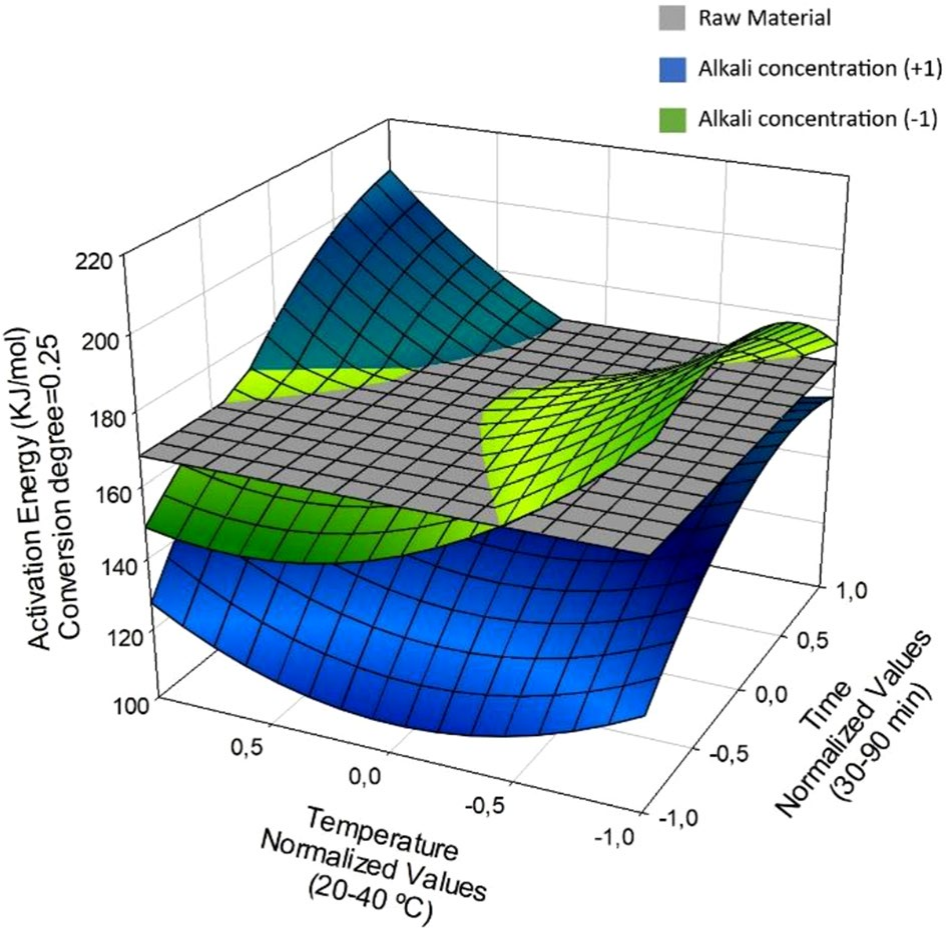
Activation energy contrast in post-CAE solid phases and raw material at α = 0.25, varying conditions.

**Figure 4:**
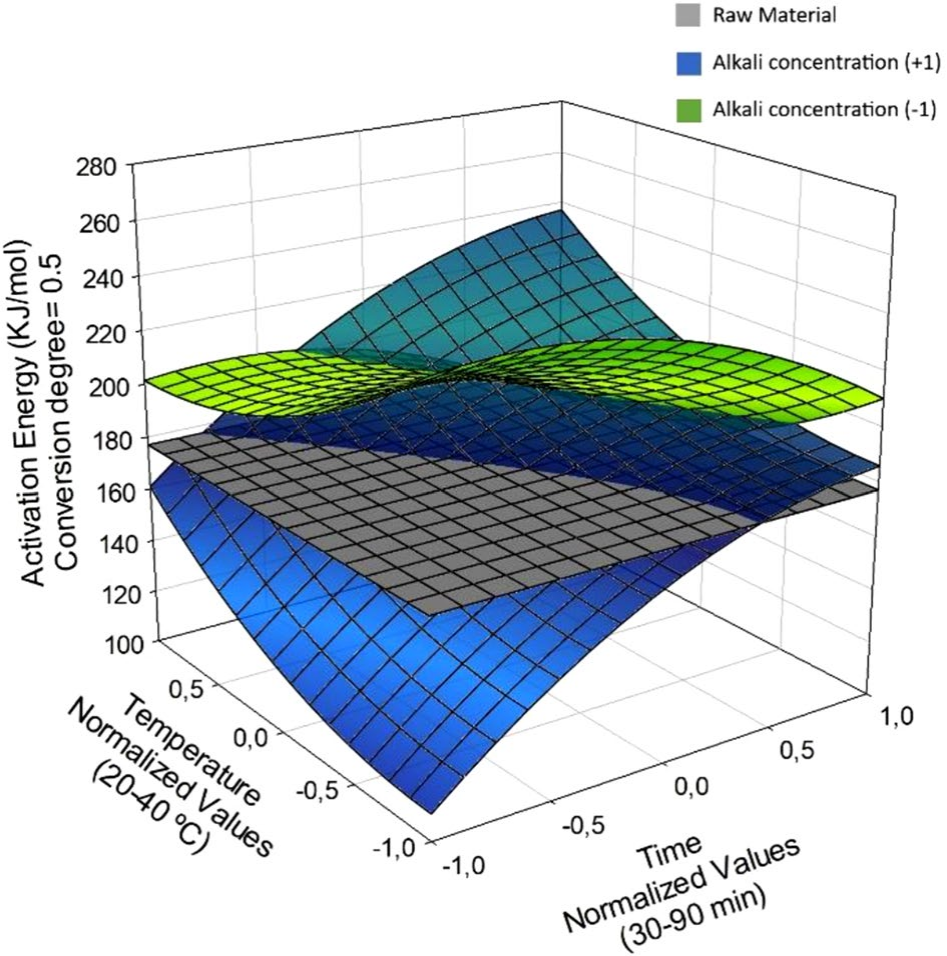
Activation energy constrast in post-CAE solid phases and raw material at α = 0.50, varying conditions.

**Figure 5:**
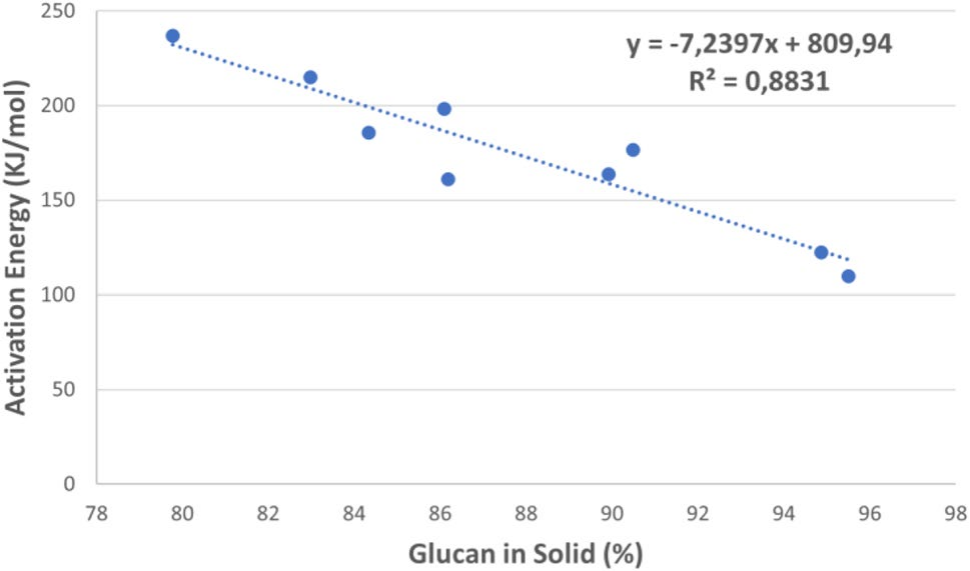
Activation energy change with post-CAE solid phases’ glucan content at α = 0.5.

Figures 3, 4, 5 show the response surfaces for the models, which were used to identify the ranges of the independent variables leading to the lowest activation energy for the post-CAE solid phases relative to the raw material. Surfaces were plotted at two different levels of alkali concentration, which was the individual independent variable most strongly influencing *E*_a_. For easier viewing and understanding, the activation energy for the initial, untreated biomass was plotted on a fixed plane so it would not change along the axes representing the CAE operating conditions.

Figure 3, which corresponds to Eq. (11), shows the variation of *E*_a_ for the post-CAE solid phases as a function of temperature and time at the highest and lowest alkali concentration studied. As can be seen, *E*_a_ was independent of both variables throughout their operation ranges. Also, at α = 0.25 it was lower for the post-CAE solid phases than it was for the raw material. Thus, *E*_a_ spanned the range 108.4–205.1 kJ/mol, which is similar to those for hemicelluloses reported by other authors. Some overall kinetic methods for hemicellulose pyrolysis have provided *E*_a_ values from 100 to 220 kJ/mol^62^.

It should be noted that *E*_a_ was lowest for the post-CAE solid phases resulting from treatments at high alkali concentrations and short times, the temperature having little effect on the results.

Figure 4 shows the variation of *E*_a_ with the treatment temperature and time at the highest and lowest alkali concentration —the most influential variable— at α = 0.5. As can be seen, *E*_a_ ranged from 109.7 to 254.3 226 kJ/mol and was largely due to degradation of the cellulose fraction. The average activation energy for cellulose is 189.2 kJ/mol^63^.

As can be seen from Fig. 5, a combination of the highest alkali concentration, lowest temperature and shortest treatment time led to the lowest *E*_a_ values relative to the raw material. On the other hand, *E*_a_ remained fairly constant at the lowest alkali level. There was strong correlation between the activation energy and the amount of glucan remaining in the post-CAE solid phase. In fact, as can be seen in Fig. 5, constructed at the highest alkali concentration, *E*_a_ decreased with increasing proportion of glucan in the solid as a result of the cellulose fraction having a lower activation energy than the polyphenolic fraction.

**Figure 6:**
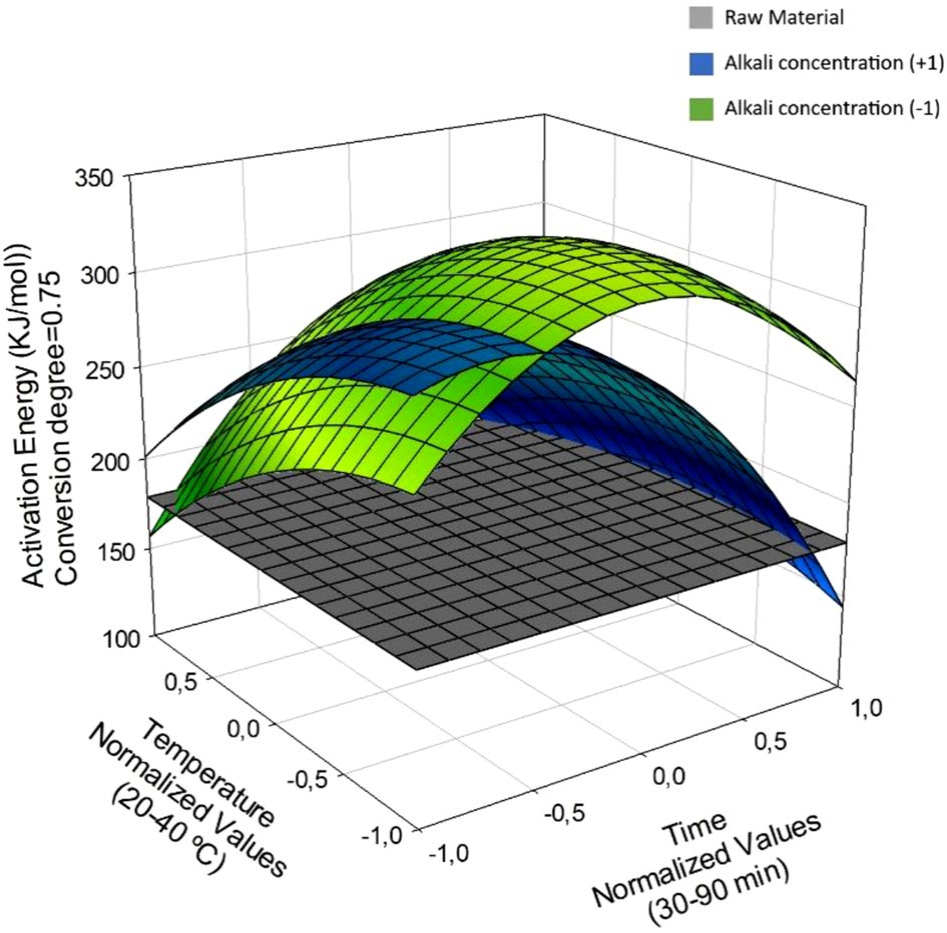
Activation energy contrast in post-CAE solid phases and raw material at α = 0.75, varying conditions.

As can be seen in Fig. 6, at α = 0.75 the activation energy for the post-CAE solid phases was higher than it was for the raw material under virtually any operating conditions and ranged from 132.9 to 328.5 kJ/mol. Cai et al.^60^ reported *E*_a_ values of 237.1–266.6 kJ/mol for lignin, whereas Murugan et al.^64^ found the isothermal activation energy for the polyphenolic fraction to be 284 kJ/mol.

### Hydrogen production

The thermogravimetric and kinetic study of the pyrolytic process was followed by assessment of the hydrogen production potential of the post-CAE solid phases on the laboratory-scale reactor described in Section "Modeling and optimization of activation energy".

The TGA curves previously obtained were used to identify the best temperature for hydrogen production. Table 5 shows the selected temperature and the maximum quantity of hydrogen generated for each post-CAE solid phase.

**Table 5:** Hydrogen production from the post-CAE solid phases.

Operating conditions (*X*_A_, *X*_*t*_, *X*_*T*_)			Temperature (ºC)	Hydrogen (vol. %)
0	0	0	309	13.26
1	1	1	305	6.44
1	1	− 1	309	19.07
1	− 1	1	313	13.69
1	− 1	− 1	310	12.76
− 1	1	1	315	10.98
− 1	1	− 1	323	8.31
− 1	− 1	1	315	7.90
− 1	− 1	− 1	303	6.63
1	0	0	315	8.65
− 1	0	0	323	12.94
0	1	0	317	8.84
0	− 1	0	315	10.17
0	0	1	318	8.86
0	0	− 1	316	11.33
Raw material			372	4.04

Independent variables: X_A_ alkali concentration, *X*_*T*_ temperature, *X*_*t*_ treatment time.

The data in Table 5 underwent multiple regression analysis as a function of the three independent variables (alkali concentration, temperature and treatment time). Similar to *E*_a_, the proportions, in volume, of maximum hydrogen obtained with each post-CAE solid phase were considered. The resulting equation was: H_2_ = 11.85 + 2.32*X*_A_—1.83*X*_*T*_ + 2.94*X*_A_^2^—2.24*X*_*T*_^2^—1.65*X*_A_*X*_*T*_—1.26*X*_*t*_*X*_*T*_ with a quadratic regression coefficient of 0.98 and a coefficient of variation less than 10%. All independent variables had Student’s *t*-values greater than 2, so they were deemed statistically significant.

**Figure 7:**
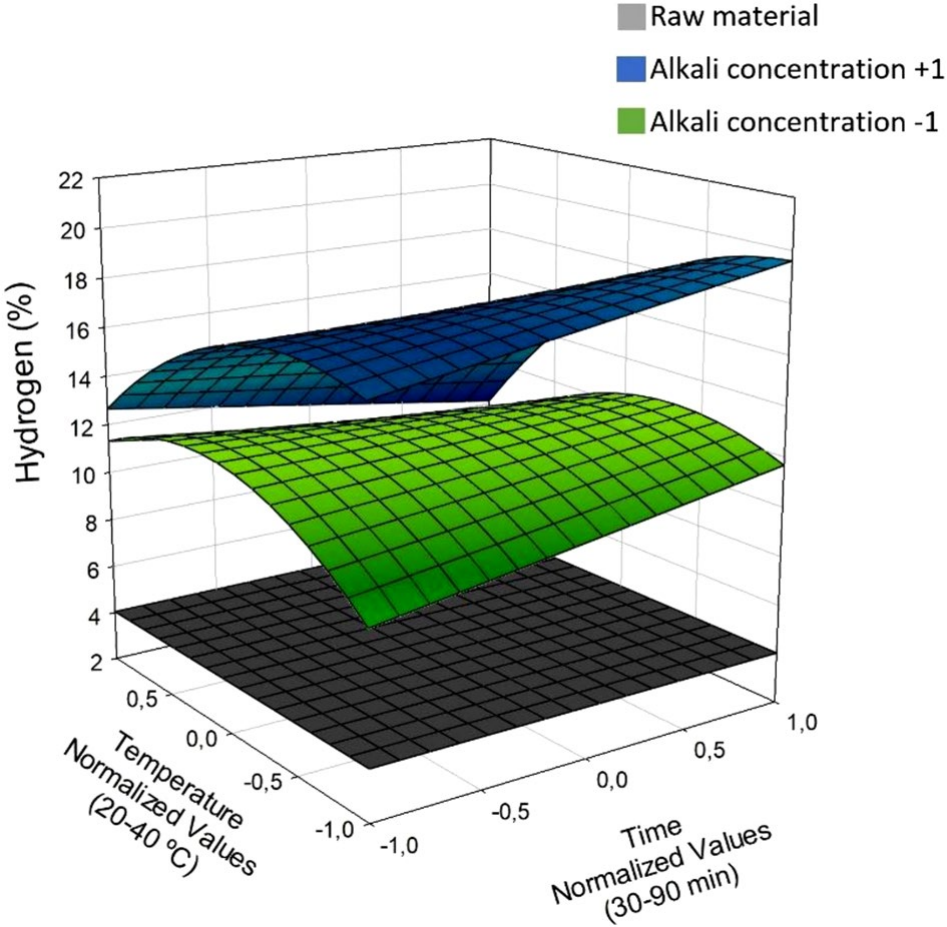
Hydrogen production variation in post-CAE solid phases and raw material at α = 0.50, diverse conditions.

Figure 7 shows the response surface for hydrogen production. As can be seen, the post-CAE solid phases, but particularly those obtained at the highest alkali concentration, produced greater amounts of hydrogen than did the raw material. On the other hand, as was apparent from Eq. (14), hydrogen production was scarcely affected by the treatment time. Finally, the effect of temperature was found to depend on the alkali concentration; thus, at the highest alkali levels, hydrogen production peaked with medium or high temperatures.

The maximum proportion of hydrogen obtained, in volume, from the post-CAE solid phases ranged from 9.15 to 19.51%, so it was up to 15.57% greater than that provided by the raw material. These results are consistent with previously reported values for species such as *Eucalyptus urograndis*, where authohydrolysis provided amounts of hydrogen up to 8% greater than the raw material^33^. Similary, the application of acid hydrolysis to *Leucaena leucocephala* resulted in increased hydrogen production^53^. In general, slow pyrolysis, like the process studied in this work, exhibits a range of hydrogen yields between 9 and 44%^65^.

## Conclusions

As shown here, ultrasound-assisted cold alkaline extraction provides liquors with increased proportions of hemicelluloses and lignin relative to the absence of ultrasound.

The composition of the raw material and the post-CAE solid phases affect their thermal degradation. In addition, the post-CAE solid phases were more reactive in the thermochemical process than was clone AF2 clone of *Populus*.

The activation energy of the post-CAE solid phases is generally lower than that of the raw material, with alkali concentration proving to be the most influential independent variable in this regard. In turn, *E*_a_ decreased with increasing proportion of glucan in the residues.

The ultrasound-assisted CAE process enhances hydrogen production, making this sequential process with CAE and pyrolysis approach a favorable choice for this feedstock valorization.

## Data Availability

All data generated or analyzed during this work are included in this published article.

## References

[1] Jiang Z, Rahman Mahmud A, Maneengam A, Nassani AA, Haffar M, The CP (2022). Non linear effect of Biomass, fossil fuels and renewable energy usage on the economic Growth: Managing sustainable development through energy sector. Fuel.

[2] Popp J, Kovács S, Oláh J, Divéki Z, Balázs E (2021). Bioeconomy: Biomass and biomass-based energy supply and demand. N. Biotechnol..

[3] Schimel D, Stephens BB, Fisher JB (2015). Effect of increasing CO _2_ on the terrestrial carbon cycle. Proc. Nat. Acad. Sci..

[4] Welfle A, Thornley P, Röder M (2020). A review of the role of bioenergy modelling in renewable energy research & policy development. Biomass Bioenergy..

[5] Nie A, Kung SS, Li H, Zhang L, He X, Kung CC (2021). An environmental and economic assessment from bioenergy production and biochar application. J. Saudi Chem. Soc..

[6] Yousaf I, Nekhili R, Umar M (2022). Extreme connectedness between renewable energy tokens and fossil fuel markets. Energy Econ..

[7] Bender TA, Dabrowski JA, Gagné MR (2018). Homogeneous catalysis for the production of low-volume, high-value chemicals from biomass. Nat. Rev. Chem..

[8] Roy S, Dikshit PK, Sherpa KC, Singh A, Jacob S, Chandra RR (2021). Recent nanobiotechnological advancements in lignocellulosic biomass valorization: A review. J. Environ. Manage..

[9] Saravanan A, Senthil Kumar P, Jeevanantham S, Karishma S, Vo DVN (2022). Recent advances and sustainable development of biofuels production from lignocellulosic biomass. Bioresour. Technol..

[10] Abraham A, Mathew AK, Park H, Choi O, Sindhu R, Parameswaran B (2020). Pretreatment strategies for enhanced biogas production from lignocellulosic biomass. Bioresour. Technol..

[11] FitzPatrick M, Champagne P, Cunningham MF, Whitney RA (2010). A biorefinery processing perspective: Treatment of lignocellulosic materials for the production of value-added products. Bioresour. Technol..

[12] García JC, Díaz MJ, Garcia MT, Feria MJ, Gómez DM, López F (2013). Search for optimum conditions of wheat straw hemicelluloses cold alkaline extraction process. Biochem. Eng. J..

[13] Yuan Z, Long J, Wang T, Shu R, Zhang Q, Ma L (2015). Process intensification effect of ball milling on the hydrothermal pretreatment for corn straw enzymolysis. Energy Convers. Manag..

[14] de Carvalho DM, de Queiroz JH, Colodette JL (2016). Assessment of alkaline pretreatment for the production of bioethanol from eucalyptus, sugarcane bagasse and sugarcane straw. Ind. Crops Prod..

[15] Abejón R (2018). A bibliometric study of scientific publications regarding hemicellulose valorization during the 2000–2016 period: Identification of alternatives and hot topics. ChemEngineering.

[16] Chen Z, Zhu Q, Wang X, Xiao B, Liu S (2015). Pyrolysis behaviors and kinetic studies on Eucalyptus residues using thermogravimetric analysis. Energy Convers. Manag..

[17] Bridgwater AV (2003). Renewable fuels and chemicals by thermal processing of biomass. Chem. Eng. J..

[18] Zhang L, Chen K, He L, Peng L (2018). Reinforcement of the bio-gas conversion from pyrolysis of wheat straw by hot caustic pre-extraction. Biotechnol. Biofuels..

[19] Kan T, Strezov V, Evans TJ (2016). Lignocellulosic biomass pyrolysis: A review of product properties and effects of pyrolysis parameters. Renew. Sustain. Energy Rev..

[20] Yang H, Yan R, Chen H, Lee DH, Zheng C (2007). Characteristics of hemicellulose, cellulose and lignin pyrolysis. Fuel.

[21] Yogalakshmi KN, Poornima Devi T, Sivashanmugam P, Kavitha S, Yukesh Kannah R, Sunita Varjani S, AdishKumar GK, Rajesh Banu J (2022). Lignocellulosic biomass-based pyrolysis: A comprehensive review. Chemosphere.

[22] Ni M, Leung DYC, Leung MKH, Sumathy K (2006). An overview of hydrogen production from biomass. Fuel Process. Technol..

[23] Buffi M, Prussi M, Scarlat N (2022). Energy and environmental assessment of hydrogen from biomass sources: Challenges and perspectives. Biomass Bioenergy.

[24] Commission European. Communication from the Commission to the European Parliament, the Council, the European Economic and Social Committee and the Committee of the Regions Youth Opportunities Initiative. 2011;

[25] Nasir Uddin M, Daud WMAW, Abbas HF (2013). Potential hydrogen and non-condensable gases production from biomass pyrolysis: Insights into the process variables. Renew. Sustain. Energy Rev..

[26] Demirbaş A (2002). Gaseous products from biomass by pyrolysis and gasification: Effects of catalyst on hydrogen yield. Energy Convers. Manag..

[27] Widyawati M, Church TL, Florin NH, Harris AT (2011). Hydrogen synthesis from biomass pyrolysis with in situ carbon dioxide capture using calcium oxide. Int. J. Hydrogen Energy.

[28] Sanchez-Silva L, López-González D, Villaseñor J, Sánchez P, Valverde JL (2012). Thermogravimetric–mass spectrometric analysis of lignocellulosic and marine biomass pyrolysis. Bioresour. Technol..

[29] Kumar JV, Pratt BC (1996). Compositional analysis of some renewable biofuels. Am. Lab. (Fairfield).

[30] Li S, Xu S, Liu S, Yang C, Lu Q (2004). Fast pyrolysis of biomass in free-fall reactor for hydrogen-rich gas. Fuel Process. Technol..

[31] Wu C, Wang Z, Huang J, Williams PT (2013). Pyrolysis/gasification of cellulose, hemicellulose and lignin for hydrogen production in the presence of various nickel-based catalysts. Fuel.

[32] Jarungthammachote S, Acharya B, Dutta A. Parametric Effect of Agricultural Residues and Woody Biomass on Hydrogen Production through Gasification Process. Proceedings of the international agricultural engineering conference Cutting edge technologies and innovations on sustainable resources for world food sufficiency: Asian Association for Agricultural Engineering. Citeseer; 2007.

[33] Loaiza JM, Palma A, Díaz MJ, Ruiz-Montoya M, García MT, García JC (2022). Effect of autohydrolysis on hemicellulose extraction and pyrolytic hydrogen production from Eucalyptus urograndis. Biomass Convers. Biorefin..

[34] González-González BD, Oliveira N, González I, Cañellas I, Sixto H (2017). Poplar biomass production in short rotation under irrigation: A case study in the Mediterranean. Biomass Bioenergy.

[35] Sixto H, Cañellas I, van Arendonk J, Ciria P, Camps F, Sánchez M (2015). Growth potential of different species and genotypes for biomass production in short rotation in Mediterranean environments. For. Ecol. Manage..

[36] Bloemen J, Fichot R, Horemans JA, Broeckx LS, Verlinden MS, Zenone T (2017). Water use of a multigenotype poplar short-rotation coppice from tree to stand scale. GCB Bioenergy.

[37] Navarro A, Facciotto G, Campi P, Mastrorilli M (2014). Physiological adaptations of five poplar genotypes grown under SRC in the semi-arid mediterranean environment. Trees – Struct. Funct..

[38] di Matteo G, Nardi P, Verani S, Sperandio G (2015). Physiological adaptability of Poplar clones selected for bioenergy purposes under non-irrigated and suboptimal site conditions: A case study in Central Italy. Biomass Bioenergy.

[39] TAPPI T264 cm-07 (2007). Preparation of Wood for Chemical Analysis.

[40] TAPPI T212 om-22. One percent sodium hydroxide solubility of wood and pulp (Test Method).

[41] TAPPI T257 cm-85 (1999). Sampling and Preparing Wood for Analysis.

[42] TAPPI T204 cm-07 (2007). Sampling and Preparing Wood for Analysis.

[43] TAPPI 211 om-02 (2002). Ash in Wood, Pulp, Paper and Paperboard: Combustion at 525°C..

[44] TAPPI T249 cm-09 (2009). Carbohydrate Composition of Extractive-Free Wood and Wood Pulp by Gas-Liquid Chromatography.

[45] TAPPI T222 om-11 (2011). Acid-Insoluble Lignin in Wood and Pulp.

[46] Montgomery DC (2003). Diseño y Análisis de Experimentos.

[47] Vyazovkin S, Sbirrazzuoli N (2006). Isoconversional kinetic analysis of thermally stimulated processes in polymers. Macromol. Rapid. Commun..

[48] Starink MJ (2003). The determination of activation energy from linear heating rate experiments: A comparison of the accuracy of isoconversion methods. Thermochim Acta..

[49] Martín-Lara MA, Ronda A, Blázquez G, Pérez A, Calero M (2018). Pyrolysis kinetics of the lead-impregnated olive stone by non-isothermal thermogravimetry. Process Safety Environ. Protect..

[50] Friedman HL (1964). Kinetics of thermal degradation of char-forming plastics from thermogravimetry. Application to a phenolic plastic. J. Polym. Sci. Part C: Polym. Symposia.

[51] Susana Lozano Calvo. Extraction of Polysaccharides in High-Yield Species Using Ultrasound. 2019.

[52] Methrath Liyakathali NA, Muley PD, Aita G, Boldor D (2016). Effect of frequency and reaction time in focused ultrasonic pretreatment of energy cane bagasse for bioethanol production. Bioresour. Technol..

[53] Loaiza JM, López F, García MT, García JC, Díaz MJ (2017). Biomass valorization by using a sequence of acid hydrolysis and pyrolysis processes. Application to Leucaena leucocephala. Fuel.

[54] Pontes R, Romaní A, Michelin M, Domingues L, Teixeira J, Nunes J (2018). Comparative autohydrolysis study of two mixtures of forest and marginal land resources for co-production of biofuels and value-added compounds. Renew. Energy.

[55] Dantas GA, Legey LFL, Mazzone A (2013). Energy from sugarcane bagasse in Brazil: An assessment of the productivity and cost of different technological routes. Renew. Sustain. Energy Rev..

[56] Sebio-Puñal T, Naya S, López-Beceiro J, Tarrío-Saavedra J, Artiaga R (2012). Thermogravimetric analysis of wood, holocellulose, and lignin from five wood species. J. Therm. Anal. Calorim..

[57] Fidalgo ML, Terron MC, Martinez AT, Gonzalez AE, Gonzalez-Vila FJ, Galletti GC (1993). Comparative study of fractions from alkaline extraction of wheat straw through chemical degradation, analytical pyrolysis, and spectroscopic techniques. J. Agric. Food Chem..

[58] Ramírez Á, García-Torrent J, Tascón A (2010). Experimental determination of self-heating and self-ignition risks associated with the dusts of agricultural materials commonly stored in silos. J. Hazard Mater..

[59] Carrier M, Auret L, Bridgwater A, Knoetze JH (2016). Using apparent activation energy as a reactivity criterion for biomass pyrolysis. Energy Fuels.

[60] Cai J, Wu W, Liu R, Huber GW (2013). A distributed activation energy model for the pyrolysis of lignocellulosic biomass. Green Chem..

[61] Vyazovkin S (2016). A time to search: Finding the meaning of variable activation energy. Phys. Chem. Chem. Phys..

[62] Zhou X, Li W, Mabon R, Broadbelt LJ (2017). A critical review on hemicellulose pyrolysis. Energy Technol..

[63] Chen D, Cen K, Zhuang X, Gan Z, Zhou J, Zhang Y (2022). Insight into biomass pyrolysis mechanism based on cellulose, hemicellulose, and lignin: Evolution of volatiles and kinetics, elucidation of reaction pathways, and characterization of gas, biochar and bio-oil. Combust Flame..

[64] Murugan P, Mahinpey N, Johnson KE, Wilson M (2008). Kinetics of the pyrolysis of lignin using thermogravimetric and differential scanning calorimetry methods. Energy Fuels.

[65] Vuppaladadiyam AK, Vuppaladadiyam SSV, Awasthi A, Sahoo A, Rehman S, Pant KK (2022). Biomass pyrolysis: A review on recent advancements and green hydrogen production. Bioresour. Technol..

